# Genetic Diversity and Population Structure Assessed Using Microsatellite (SSR) Markers from Relict Populations of *Nuphar pumila* (Nymphaeaceae)

**DOI:** 10.3390/plants12091771

**Published:** 2023-04-26

**Authors:** Claudia González-Toral, Candela Cuesta, Eduardo Cires

**Affiliations:** 1 Department of Organisms and Systems Biology, University of Oviedo, C/Catedrático Rodrigo Uría s/n, 33071 Oviedo, Spain; 2 Polytechnic School of Mieres (PSM), University of Oviedo, 3ª Planta Ala Sur. C/Gonzalo Gutiérrez Quirós s/n, 33600 Mieres, Spain; 3 Institute of Natural Resources and Territorial Planning (INDUROT), Campus de Mieres, C/Gonzalo, Gutiérrez Quirós s/n, 33600 Mieres, Spain

**Keywords:** Cantabrian Mountains, genetic diversity, Iberian Peninsula, isolation, Massif Central, microsatellites, *Nuphar*, relict populations

## Abstract

The genus *Nuphar* (Nymphaeaceae) comprises aquatic plant species inhabiting freshwater bodies of the Northern hemisphere temperate regions known as yellow water-lilies. *Nuphar lutea* and *N. pumila* are the only representatives in the European continent and present different ecologies: the former is a widespread generalist, while the latter is restricted to northern latitudes or high-altitudes due to its requirements for colder and oligotrophic waters. The Central Europe mountainous areas, the Massif Central (France) and the Cantabrian Mountains (north Iberian Peninsula) harbor relict isolated *N. pumila* populations endangered by eutrophication and hybridization with *N. lutea*. We aim to detect hybridization processes in the Massif Central and Cantabrian Mountains populations and compare the genetic diversity of *N. pumila* in the relict populations of Central Europe by using microsatellite (SSR) markers. No evidence of hybridization was found in the Iberian population, whereas the admixture between *N. pumila* and *N. lutea* in the Massif Central populations could be due to hybridization or ancient introgression. Our current knowledge would benefit from genetic diversity studies focusing on both species throughout their distributional range. The Iberian and Massif Central *N. pumila* populations were genetically distinct, representing two different clusters from other relict populations, with low genetic diversity and a genetic boundary within Central Europe.

## 1. Introduction

The Nymphaeaceae genus *Nuphar* Sm. (1809) comprises 11 perennial herbaceous aquatic species, commonly known as yellow water-lilies, which inhabit freshwater bodies of the Northern hemisphere temperate regions [1,2,3,4]. In the European continent, only two representatives of this genus can be found: *Nuphar lutea* (L.) Sm. (1809) and *Nuphar pumila* (Timm) DC. (1821) [3,5]. These closely related species, which have been reported to be genetically and morphologically similar [1,6], display different ecological preferences linked to factors, such as water temperature, altitude and water depth, that determine their distribution ranges [5,7,8,9]. Thus, *N. lutea* is a more generalist species that inhabits warmer waters and tolerates higher salinity, moderately eutrophic habitats and water movements, while *N. pumila*—which is considered to be part of the “boreal–alpine glacial relicts”—prefers higher altitudes at southern latitudes (e.g., the Alps, Jura, and Vosges), not very deep colder acid waters of mountain lakes, and oligotrophic and dystrophic habitats [3,5,7,8,9,10].

Both species are distributed through the lakes of Eurasia, being widespread in northern latitudes [6,11,12,13]. However, while *N. lutea* occupies vast territories in southern latitudes extending from the British Islands and the Mediterranean basin in the West—including the Balkans and most the Italic and Iberian Peninsulas—to China and Japan in the East [3,4,14,15,16,17], the southern distribution of *N. pumila* comprises a series of relict populations confined to the British Islands and European mountainous areas of the Alps, the French Massif Central and the Cantabrian Mountains (North Spain) in the West, and the northern lakes of Mongolia, east China and Japan in the East [3,4,6,9,14,15,18,19,20,21,22]. In recent decades, many *N. pumila* populations in their natural range have been reported to have disappeared due to hybridization with *N. lutea*—which generates the fertile hybrid *Nuphar* × *spenneriana* Gaudin—and *Nuphar japonica* DC. (1821) in Japan; the degradation of their habitat due to human activity (e.g., forestry clearance or leisure activities); the eutrophication of water (i.e., increase in water hardness and humid compounds); or the decrease in water levels [5,10,19,23,24]. This situation is especially dramatic in the southernmost populations of Western and Central Europe, which inhabit the Alps and their surrounding mountainous systems, the Massif Central and the Cantabrian Mountains, where the isolation of these relict populations with high conservational interest corresponds to the previously described threats [4,9,10,25]. These factor have led to the inclusion of *N. pumila* in several European red lists with different degrees of concern, such as Least Concern (LC) at a continental and global scale in the European red list of vascular plants [26] and The IUCN Red List of Threatened Species [27]; Vulnerable (VU) in France at a national level and in the French region of Lorraine [28]; Endangered (EN) in Belarus, Poland, Switzerland, Spain and the French region of Franche-Comté [10,28,29,30,31,32]; and Critically Endangered (CR) in Austria, the Czech Republic, Germany and the French Regions of Alsace and Auvergene [10,28,33,34,35].

The development of primers for the use microsatellite markers (also known as Simple Sequence Repeats (SSRs)) in *Nuphar* by Ouborg et al. [36] has allowed the detection of hybridization (i.e., the presence of *Nuphar* × *spenneriana*) and introgression processes between *N. pumila* and *N. lutea* in Central Europe [5,9] and the discard of its presence in England [15]. In this sense, the disappearance of populations in recent decades reported in Central Europe has been correlated with the displacement by the hybrid *Nuphar* × *spenneriana* using microsatellites, thus suggesting that the presence of this hybrid is one of the major threats for the survival of *N. pumila* in the area [5,9,10]. However, a recent study by Volkova et al. [6] based on nuclear and plastid makers, which focused on the east European and central and east Asian populations, suggest that the hybridization between the two species is not as common as previously thought based on morphology, e.g., [3,15,37]. Moreover, *Nuphar* × *spenneriana* has been suggested to be capable of displacing either of its parental species depending on the scenario [6,8].

Regarding the *N. pumila* populations isolated from Western Europe, a recent molecular study based on an Internal Transcribed Spacer (ITS) and the plastid markers trnL-F and trnH-psbA, which focused on the Massif Central and the Cantabrian Mountains, did not find evidence of hybridization with *N. lutea*; suggesting that other factors—such as desiccation of water bodies—could represent a greater threat in southwest Europe [4]. Nevertheless, as highlighted by Volkova et al. [6], more sensitive molecular markers, such as microsatellites, should be employed to corroborate this absence of hybridization between the two species in southwest Europe. Furthermore, given the isolation of the Iberian population, which is separated by more than 800 km from the nearest known population, and the tendency of *N. pumila* to reproduce via clonal reproduction, more genetic information is needed to correctly implement conservation plans in order to guarantee the mid- and long-term viability of this population [4,20,25]. In this context of isolated relict populations that could be hybridizing with *N. lutea*, we aim to use SSRs to (1) determine whether there is hybridization between the Iberian Peninsula and Massif Central *N. pumila* populations and their neighboring *N. lutea* populations, and (2) to compare the genetic composition of the Iberian Peninsula and Massif Central *N. pumila* populations with the populations in the Alps and their surrounding areas in order to determine the number of conservational units, their genetic affinity and the spatial genetic patterns existing between the three *N. pumila* refugia.

## 2. Materials and Methods

### 2.1. Sampling

**Figure 1:**
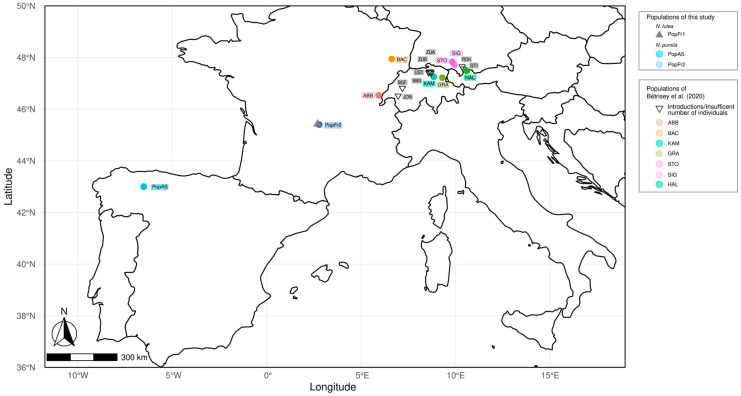
Geographic location of the relict isolated populations of *Nuphar pumila* and *N. lutea* used for this study. The populations sampled for this study are represented as blue circles (*N. pumila*) and grey triangles (*N. lutea*). The populations of *N. pumila* from Bétrisey et al. [9] used in this study are shown as colored circles (population samples used for the population diversity analyses) and inverted triangles (population samples not used for the population diversity analyses).

Fresh foliar tissues and voucher materials from 43 individuals belonging to three different isolated populations of *Nuphar* (PopAs, PopFr1 and PopFr2) was collected for this study and deposited in the Herbarium of the University of Oviedo (FCO) (see Figure 1). Two of these isolated populations are found in the French Massif Central (south-west France): the *Nuphar lutea* population of the Lac de Laspiadales (Auvergne–Rhône–Alpes, France) (PopFr1) and *N. pumila* population of the Lac de La Landie (Auvergne–Rhône–Alpes, France) (PopFr2). The third isolated population is the north Iberian *N. pumila* population of the Laguna de Reconcos (Cantabrian Mountains, Asturias, Spain). The samples were kept in silica gel during transport and before DNA extraction.

### 2.2. DNA Extraction and Amplification

The genomic DNA of the sampled individuals was extracted using the Qiagen DNeasy^®^ Plant Minikit (Qiagen Inc., Valencia, CA, USA). The extracted DNA was preserved at −20 °C before utilization. A pilot study was conducted using microsatellites or Single Sequence Repeats (SSRs) in *Nuphar* [36], followed by testing and evaluation to select the most successful ones [38]. The use of standardized protocols and primers allowed us to obtain a high level of repeatability. The PCR protocol and the primers NLGA7 and NLTG/GA1 were used to amplify these two different microsatellites, with the reverse primers being marked with the fluorophores FAM and HEX, respectively (for more detail, see Table S1). The amplicons were sequenced in the facilities of STABVIDA (Lisboa, Portugal) using capillary electrophoresis, with the fluorophore ROX as the standard.

### 2.3. Genetic Diversity, Genetic Structure and Admixture of Iberian and French N. pumila and N. lutea Isolated Populations

The sequences were visualized using GeneMarker^®^ [39] and coded numerically according to the number of repetitions. Once the data matrix was generated, several parameters focusing on genetic diversity and structure were estimated using GeneALEx 6.5 [40] to assess the genetic diversity within and between populations. The genetic structure of these 3 populations (PopAS, PopFr1 and PopFr2) was inferred via the estimation of Wright’s F-statistics and other population parameters [41,42,43]. Thus, the number of observed alleles per locus (*N_A_*), the effective number of alleles per locus (*N_E_*), the expected heterozygosity (*H_E_*) and the observed heterozygosity (*H_O_*) were estimated for each of the three different populations. Additionally, the Shannon Information Index (*I*) (Shannon 1948), which measures variation among populations, and the standardized coefficient of genetic differentiation (*G″_ST_*) [44,45] using 9999 permutations were estimated as measures of genetic structure and differentiation.

The divergence between the populations was estimated by calculating the pairwise Nei’s standard genetic distance (D) [46,47], which measures the divergence of the populations per locus and increases proportionally to the population divergence time as it takes into account the effects of mutation and genetic drift; this was followed by the pairwise *F_ST_* [48], which calculates the genetic divergence among populations.

An Analysis of Molecular Variance (AMOVA) [49,50,51,52], a statistical test capable of generating hierarchical partition of genetic variance, was conducted using GeneALEx 6.5. The analysis used the *F_ST_* to calculate the genetic distance between the populations and 9999 random permutations to estimate the Fixation Index (*F_ST_*); the Inbreeding Coefficient (*F_IS_*), which measures inbreeding due to differentiation within the subpopulations; the Total Inbreeding Coefficient (*F_IT_*), which measures inbreeding due to differentiation within and among the subpopulations; and the number of effective migrants per generation (*Nm*) [41,42,43] using the Peakall et al. [52] and Weir and Cockerham [53] methods. The statistical significance (*p*-value) of the AMOVA analysis, which null hypothesis is the absence of difference between populations (*F_ST_* = 0), was set at *p*-value ≤ 0.05. The genetic similarity between populations was also determined using the distance-based method Principal Coordinate Analysis (PCoA), following the method of [54] as implemented in GeneALEx 6.5. The two axes that best explained the genetic diversity were graphically represented using the ggplot2 v 3.4.0 R package [55].

We used the Bayesian methods implemented in STRUCTURE v.2.3.4 [56,57] to determine the total number of genetic clusters within these *Nuphar* populations. The analysis consisted of 10 independent runs for each K value, which consisted of a burn-in fraction of 10.000 and 100.000 Markov Chain Monte Carlo (MCMC) repetitions while considering the independence of allele frequencies and the admixture model. Since individuals from 3 populations were tested, the number of assumed clusters (K) was set to 9. Since the number of individuals sampled from each population was unequal, the most adequate value of K was determined using the Evanno method [58] and the Puechmaille method [59] as implemented in the online software StructureSelector [60,61]. The Evanno method uses the rate of change of probability of the data given successive values of K (i.e., the statistic ∆K) to determine the most adequate K value [58]; however, the use of this method alone has proven to be problematic when applied to unequal sampling [59]. Hence, we followed the recommendation to use the Puechmaille method [59,60], which calculates four different estimators for each K value (median of means (MedMeaK), maximum of means (MaxMeaK), median of medians (MedMedK) and maximum of medians (MaxMedK)) based on the mean membership coefficient of a cluster (Q) of individuals belonging to a predefined population [59]. The populations were predefined based on their sampling location, while the two different mean membership coefficient thresholds were set at 50% and 80% (Q = 0.5 and Q = 0.8), following the recommendation of Puechmaille [59]. Finally, the median-based estimators (MedMeaK and MedMedK) were favored when using the Puechmaille method as these are less prone to overestimate K values [59]. The STRUCTURE results with the most adequate K values were graphically represented using a barplot generated in CLUMPAK as implemented in StructureSelector [62,63].

### 2.4. Meta-Analysis of the Genetic Diversity and Spatial Structure of the Glacial Relict European Populations of Nuphar pumila

#### 2.4.1. Cluster and Genetic Diversity Analyses

The NLGA7 and NLTG/GA1 data collected by Bétrisey et al. [9] from other European *N. pumila* populations were combined with our data to compare the observed genetic diversity and structure of the south-west European isolated *N. pumila* population with the *N. pumila* populations belonging to the south-east European limit of distribution. Since the main aim of these analyses was to assess the *N. pumila* population, the reintroduced populations of the Bétrisey et al. [9] dataset and the individuals detected and labeled as hybrids were excluded from the analyses. Additionally, individuals which were not labeled as hybrids but belonged to populations in which both species or other hybrids were detected and presented 3 or 4 alleles for the marker NLGA7 were also removed from the matrices as they were considered putative hybrids. The resulting dataset contained data from individuals belonging to 17 different *N. pumila* different populations (see Figure 1) and was used to perform the STRUCTURE analyses consisting of a burn-in fraction of 10.000 and 100.000 MCMC repetitions while considering the independence of allele frequencies and the admixture model. Since the dataset included individuals from 17 different populations, the number of assumed cluster (K) was set to 22. Both the Evanno and the Puechmaille methods [58,59], as implemented in StructureSelector, were used to determine the most adequate K value, following the same criteria as in the previous analyses. Additionally, the admixture coefficients of each population generated by CLUMPAK (clumppPopFile) based on the most adequate K value were used to graphically represent the population admixture.

The genetic diversity of the isolated south-east and south-west European *N. pumila* populations was assessed using a dataset that included at least 8 *N. pumila* individuals after the hybrids and putative hybrids had been removed, in order to avoid the effect of small population size. The genetic diversity of the populations of this dataset was evaluated by calculating the number of observed alleles per locus (*N_A_*), the effective number of alleles per locus (*N_E_*), the expected heterozygosity (*H_E_*), the observed heterozygosity (*H_O_*), the number of different genotypes (*MLG*), the expected number of different genotypes (*MLGE*), the percentage of polymorphic loci and the number of private alleles for each population using the GeneALEx 6.5 and Poppr v 2.9.3 R package [64]). The Shannon Information Index (*I*) [65] and the pairwise *F_ST_* estimated based on the method of Weir and Cockerham [53] were used to evaluate the genetic differentiation and genetic distance among the populations using the GeneALEx 6.5 and hierfstat R package v.0.5.11 [66].

The genetic structure of the isolated south-west European *N. pumila* populations was complemented by performing a PCoA [54] in GeneALEx 6.5 and a Discriminant Analysis of Principal Components (DAPC) [67], which allows the detection of the underlying structure behind clusters detected by clustering analyses, in the Adegenet v. 2.1.8 R package [68]. In the case of the DAPC, the most adequate number of PCs that should be retained was assessed by using the α-score, after which the DAPC was performed [67]. The membership probabilities of the individuals belonging to each population were also estimated. Additionally, a snapclust analysis, a clustering method based on Maximum Likelihood (ML) which uses the Expectation–Maximization algorithm for estimation [69], was performed using Adegenet v. 2.1.8. The number of PCs to be retained for this analysis was indicated by the α-score of the DAPC, while the number of clusters was determined using the find.cluster() function. The maximum number of iterations was set to 10,000, with the Ward algorithm being used to define the initial groups and the k-means algorithm being run 50 times in order to define the starting point. The relationships between the individuals of the different populations were also analyzed by generating a network based on Neighbor Joining (NJ) using a dissimilarity matrix estimated with 30.000 in DARwin 6.0.021 [70,71,72] and a Minimum Spanning Network (MSN) dendrogram based on a Bruvo distance matrix (bruvo.msn() function) in the poppr R package [73].

#### 2.4.2. Spatial Analyses and Boundary Detection

In order to determine the relevance of geographical distance in the observed genetic patterns, various tests that incorporated geographical data were conducted. Given the geographical distance that separated the studied *N. pumila* populations, the isolation by distance (*IBD*) was tested using the means of a Mantel test [74] as implemented in the ade4 v. 1.7.20 R package [75], which tested the statistical relationship between geographical distance and genetic distance. The genetic distance was estimated by calculating the pairwise *F_ST_* based on the method of [53] in hierfstat, while the pairwise geographical distance was estimated using the geodesic methods [76] as implemented in the geodist v.0.8.5 R package [77]. The statistical significance of the relationship between these two matrixes was assessed by computing 9999 permutations.

The potential spatial patterns of the observed genetic variability were assessed using a spatial explicit multivariable method, the spatial Principal Component Analysis (sPCA), as implemented in Adegenet v.2.1.8 [78]. For this purpose, two different connection networks were generated: (1) a connection network in which the Iberian population was completely disconnected from the rest of the populations (based on the geographical distance separating this population from its nearest neighbor), and (2) a connection network that completely disconnected the Iberian and Massif Central populations from the Alps populations. The number of positive and negative eigenvalues for each of the *sPCA* analyses was determined by performing the Monte Carlo tests for Local and Global structure using 9999 permutations [78].

The potential boundaries between the studied populations were detected using the Monmonier algorithm [79], which seeks the highest distance between neighbors of a network, as implemented in Adegenet v.2.1.8 for genetic data [80]. The two different networks created for the sPCA analyses were used for these analyses as well.

## 3. Results

### 3.1. Genetic Diversity in Iberian and Southern French Populations

The NLGA7 and NLTG/GA1 alleles found in the *N. pumila* and *N. lutea* south-west European isolated populations ranged from 100 to 152 repetitions and from 115 to 172 repetitions, respectively (see Table S2 for more detail). The genetic diversity parameters of the populations (see Table 1) indicated that the Massif Central isolated population of *N. pumila* had a higher number of alleles per locus and a higher number of effective alleles per locus (PopFr2: *N_A_* = 7.000 ± 1.000 and *N_E_* = 3.440 ± 0.560) than the isolated population of *N. pumila* and *N. lutea* from the north Iberian Peninsula and the Massif Central (PopAS: *N_A_* = 4.500 ± 1.500 and *N_E_* = 1.722 ± 0.301; PopFr1: *N_A_* = 4.000 ± 3.000 and *N_E_* = 1.624 ± 0.624), whereas the French and the Iberian isolated *N. pumila* populations exhibited a higher number of private alleles (PopFr2: Private alleles = 4.000; PopAS: Private alleles = 2.000) than the *N. lutea* population (PopFr1: Private alleles = 0.500). The isolated *N. lutea* population of the Massif Central had a fixed allele at the locus NLTG/GA1 (PopFr1: NLTG/GA1 = 140, %*P* = 0.50), while the other two populations had no fixed allele (PopAs: %*P* = 1.00, PopFr2: %*P* = 1.00) (see Table S3). The expected heterozygosity was similar to the observed heterozygosity for the French *N. lutea* and *N. pumila* populations (PopFr1: *H_E_* = 0.278 ± 0.278 and *H_O_* = 0.250 ± 0.250; PopFr2: *H_E_* = 0.701 ± 0.049 and *H_O_* = 0.792 ± 0.042), while the observed heterozygosity was lower than the expected value in the case of the north Iberian isolated *N. pumila* population (PopAS: *H_E_* = 0.401 ± 0.105 and *H_O_* = 0.287 ± 0.237). Regarding genetic diversity, both the fixation index and the Shannon Information Index revealed that the Massif Central isolated *N. pumila* population was the most diverse (PopFr2: *I* = 1.480 ± 0.169), followed by the north Iberian *N. pumila* population (PopAs: *I* = 0.774 ± 0.238) and the Massif Central isolated *N. lutea* population (PopFr1: *I* = 0.630 ± 0.630). The *G″_ST_* indicated a high differentiation between the three populations, while the mean number of migrants per generation did not reach one individual (*G″_ST_* = 0.808; *Nm* = 0.515 ± 0.272). 

**Figure 2:**
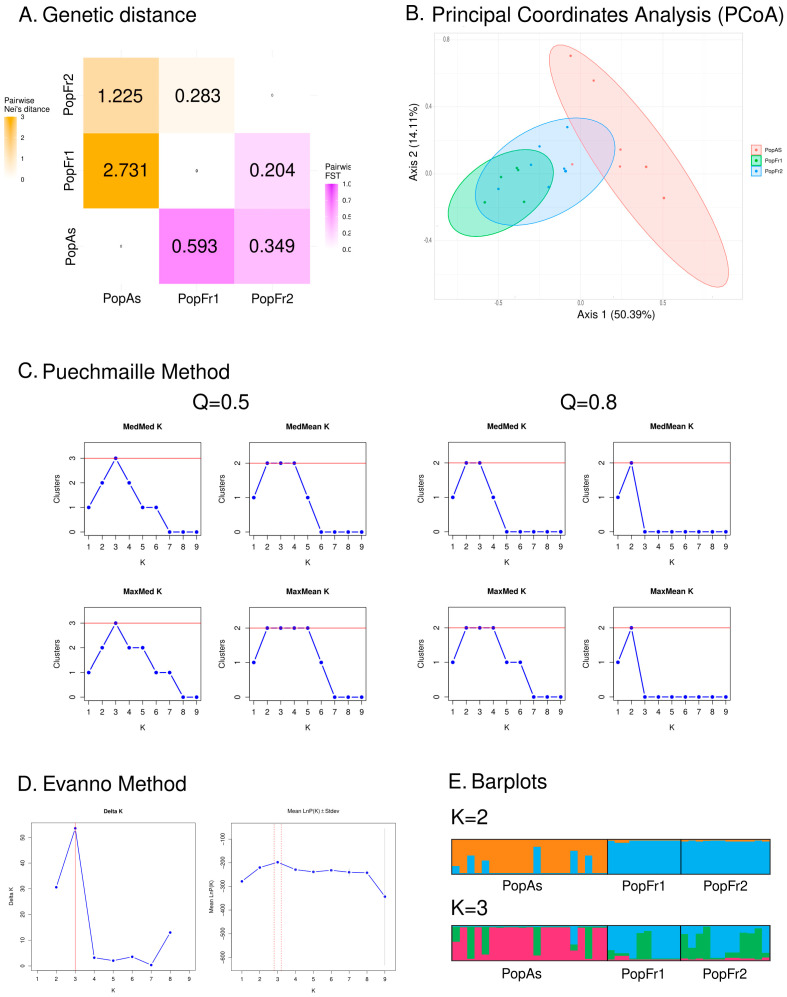
Results of the microsatellite-based analyses of the genetic diversity and structure of Iberian and south-west French isolated populations. (**A**) Heatmap based on the retrieved values of the genetic distance among populations based on the Nei’s distance (orange) and the Fixation index (*F_ST_*) (purple). (**B**) Graphical representation of the Principal Coordinate Analysis (PCoA) using the two main axes, which explain the majority of the observed genetic similarity. (**C**) Graphical representation of the four estimators (MedMeaK, MaxMeaK, MedMedK and MaxMedK) used by the Puechmaille method to determine the most adequate K value. The analyses based on the two values set for the mean membership coefficient of a cluster (Q) are represented. (**D**) Graphical representation of delta K analyses used in the Evanno method. (**E**) Barplots of the STRUCTURE results of the two most adequate K values as determined by the Evanno and Puechmaille methods.

The pairwise measurements of genetic differentiation, as estimated by the fixation indexes and the Nei distance (see Figure 2A), revealed that the north Iberian *N. pumila* population and the isolated *N. lutea* population of the Massif Central were the most different pair of populations (PopAs-PopFr1: *F_ST_* = 0.593, *D* = 2.731), while the Massif Central pair was the less genetically differentiated (PopFr2-PopFr1: *F_ST_* = 0.204, *D* = 0.283). The isolated *N. pumila* populations had a moderate level of differentiation (PopAs-PopFr2: *F_ST_* = 0.349, *D* = 1.225). The axes of the PCoA analysis (see Figure 2B), which explained a total of 64.50% of the data variability (Axis 1 = 50.39% and Axis 2 = 14.11%), revealed the existence of two clusters: one for the north Iberian *N. pumila* population and another for the French *N. pumila* and *N. lutea* populations, which overlapped. The AMOVA analysis (see Table 2) indicated that the variation within individuals (*F_IT_* = −0.511, *p* < 0.001) explained 49% of the observed genetic differences, followed by the variation among populations (*F_ST_* = 0.427, *p* < 0.001), which explained 43% of the observed variation, and the variation among individuals (*F_IS_* = −0.148, *p* = 0.018), which explained 8% of the variation. This indicated that the variation among the populations outweighed the variation within the populations.

Regarding the STRUCTURE analyses, the Puechmaille less restrictive analysis (Q = 0.5) and the Evanno methods indicated that the most adequate K value was K = 3, while the more restrictive Puechmaille method analysis (Q = 0.8) indicated that the most adequate K values were K = 2 and K = 3, as the median-based estimators (MedMeaK and MedMedK) indicated this interval (see Figure 2C,D). Thus, the most adequate K values ranged from two to three. The barplot representation of both values revealed that, in both cases, the north Iberian isolated *N. pumila* population was dominated by an exclusive cluster, while the French populations were both dominated by the same majority cluster (see Figure 2E).

### 3.2. Meta-Analysis of Genetic Diversity and Spatial Structure

#### 3.2.1. Cluster and Genetic Diversity Analyses

A total of 188 individuals belonging to 17 different Southern and Central European relict populations of *N. pumila* were studied using the STRUCTURE cluster analyses. This group of populations comprised one population from the north of the Iberian Peninsula (PopAs), one population from the Massif Central (PopFr2), one population from Jura (ABB), two populations from the west Swiss Plateau (BGF and JON), one population from Vosges (BAC), six populations from the east of the Swiss Plateau (GRA, KAM, UST, ZUA, ZUB and WAN), four populations from the German Alps (STO, SIG, ROH and STI) and one population from the east of the Alps found in Austria (HAL).

**Figure 3:**
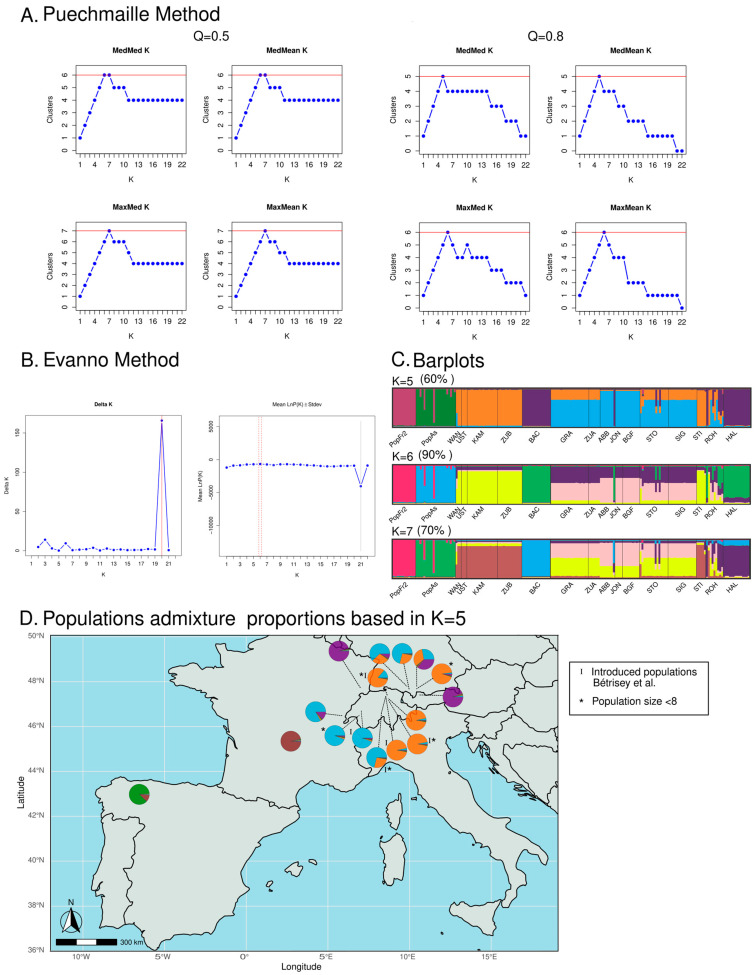
(**A**) Graphical representation of the four estimators (MedMeaK, MaxMeaK, MedMedK and MaxMedK) using the Puechmaille method to determine the most adequate K value. The analyses based on the two values set for the mean membership coefficient of a cluster (Q) are represented. (**B**) Graphical representation of delta K analyses used in the Evanno method. (**C**) Barplots of the STRUCTURE results of the two most adequate K values as determined by the Evanno and Puechmaille methods. (**D**) Graphical representation of the admixture populations based on the K = 5 STRUCTURE analysis results [9].

The Puechmaille method, which is less sensitive to uneven sampling than the Evanno method, suggested several value candidates to be the most adequate K value for the STRUCTURE analyses of the isolated European *N. pumila* populations (see Figure 3A,B). On the other hand, the Evanno method also suggested several values (K = 3, K = 6 and K = 20), with K = 20 being the value with the highest ∆K score (see Figure 3A). The latter K-value result was not supported by any of the Puechmaille method analyses, which established a range of adequate K values between K = 5 and K = 7 (Puechmaille method Q = 0.5: K = 6 and K = 7; Puechmaille method Q = 0.8: K = 5 and K = 6), therefore suggesting that K = 20 should not be considered (see Figure 3B). Hence, the most adequate values of K ranged between K = 5 and K = 7, with K = 5 being the more conservative estimation as this was the value retrieved by the median-based statistics (MedMeaK and MedMedK) for the most restrictive Puechmaille method analysis (Q = 0.8), and K = 6 was the value whereby both the Evanno method and the median-based statistics agreed when Q = 0.5. The barplot representation of the admixture coefficients of the individuals of each population revealed that the individuals from the north Iberian (PopAs) and the Massif Central isolated populations of *N. pumila* (PopFr2) belonged to two genetic clusters, which were different from the Central European populations’ clusters (see Figure 3C). The individuals of the south-west French population belonged mostly to one cluster that was shared with some of the Iberian individuals, with the Iberian population being represented as a majority private cluster. This happened with all the different K values that fell within the range of the most adequate K values using the different methods of estimation. On the other hand, the different K values retrieved affected the cluster distributions of the west and the east Swiss Plateau populations, the German Alps populations and the Jura population: when K = 5, the individuals of the east Swiss Plateau populations of GRA and ZUA and the German Alps populations of STO, SIG and ROH belonged to both the cluster of the east Swiss Plateau populations and the German Alps population of STI and the cluster of the east Swiss Plateau populations and the Jura population, whereas with higher values of K, the east Swiss Plateau populations and the German Alps population of STI belonged to a separate cluster, while the rest of the cited populations presented individuals that belonged to their own two clusters (see Figure 3C,D). Similarly, the relationship between the Vosges population (BAC) and the Austrian population (HAL) was also affected by the different values of K: when K = 7, these two populations did not share the same majority cluster. The genetic diversity analyses, which were restricted to non-introduced populations with at least 8 *N. pumila* individuals (when the hybrids and putative hybrids had been removed), focused on 131 individuals belonging to 9 different populations (see Table 3). These nine populations represented all the different clusters found in the STRUCTURE analyses at any given K value (see Figure 3C,D).

The different allelic compositions of the Iberian and south-west French isolated populations for both markers when compared to the Central European populations are presented in Table 3. 

**Figure 4:**
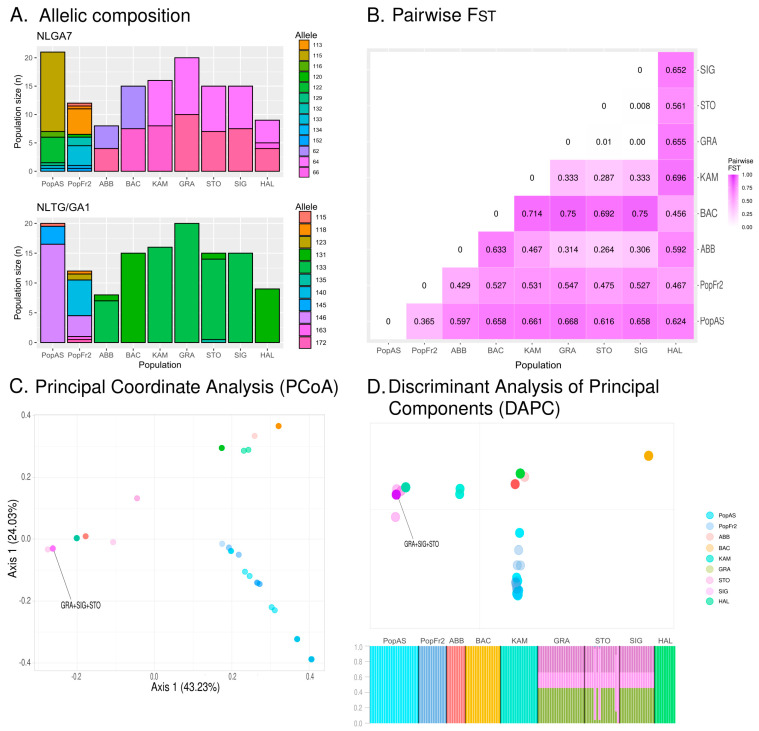
(**A**) Allelic compositions of the isolated south European *N. pumila* populations based on genetic diversity and spatial analyses. (**B**) Pairwise *F_ST_* of the studied isolated south European *N. pumila* populations. (**C**) Results of the Principal Coordinate Analysis (PCoA) of the south European *N. pumila* populations. Completely overlapping dots are indicated. (**D**) The graphical representation corresponds to the results of the Discriminant Analysis of Principal Components (DAPC) of the south European *N. pumila* populations retaining 6 Principal Components (PCs), while the barplot represents the membership probabilities of the individuals belonging to each population obtained during the DAPC. Completely overlapping dots are indicated in the graphical representation.

The north Iberian and the south-west French isolated *N. pumila* populations presented a higher number of alleles (*N_A_*) and a higher effective number of alleles (*N_E_*) than the isolated population from the Central European relict populations. The latter had *N_A_* ranging from 1.500 in the case of the populations of Vosges, east Swiss Plateau and eastmost German Alps to 2.500 in the case of westmost German Alps population (BAC, KAM, GRA and SIG: *N_A_* = 1.500 ± 0.500; STO: *N_A_* = 2.500 ± 0.500), and N_E_ ranging from 1.500 in the Vosges, east Swiss Plateau and eastmost German Alps populations to 1.727 in the case of the Austrian Alps population (BAC, KAM, GRA and SIG: *N_E_* = 1.500 ± 0.500; HAL: *N_E_* = 1.727 ± 0.727). The Iberian and the Massif Central populations did not present fixed alleles at any locus, with these populations having more private alleles (NLGA7: PopFr2-Private alleles = 6.000 and PopAS-Private alleles = 4.000; NLTG/GA1: PopFr2-Private alleles = 5.000 and PopAS-Private alleles = 2.000) for both markers than the Central European populations, where only two populations presented private alleles (NLGA7: HAL-Private alleles = 1.000; NLTG/GA1: STO-Private alleles = 1.000). Some of the Central European populations presented fixed alleles at the locus NLTG/GA1: the east Swiss Plateau populations (KAM and GRA) and eastmost German Alps population of SIG had 133 fixed alleles (KAM, GRA and SIG: %*P* = 0.500), and the Vosges and the Austrian Alps populations of BAC and HAL had 131 fixed alleles (BAC and HAL: %*P* = 0.500). The graphical representation of the allelic composition of all these populations (see Figure 4A) also reveal that the north Iberian and Massif Central *N. pumila* populations have very different compositions from that of the central Europe populations, which present slight differences among each other. In this sense, the north Iberian and the south-west French isolated *N. pumila* populations had both a higher number of different genotypes (PopAs: MLG = 7; PopFr2: MLG = 9) and a higher estimated number of different genotypes at the lowest common populations size (PopAs: MLGE = 4.62 ± 0.962; PopFr2: MLGE = 7.82 ± 0.649) than the Alps *N. pumila* population with a similar populations size (see Table 3). Regarding the heterozygosity of the populations, the expected heterozygosity (*H_E_*) was higher in the north Iberian and Massif Central populations than in the Central European populations. The lowest observed heterozygosity (*H_O_*) was that of the north Iberian population. The Massif Central population was a special case among the latter, as the Standard Error (SE) of this population was lower than in the other populations, which presented high SE values. The Shannon Information Index (*I*) also showed higher values for the north Iberian and south-west French populations than for the Alps populations, which ranged from 0.347 (with high SE values) in the case of the populations of the Vosges, the east Swiss Plateau and the eastmost German Alps populations to 0.540 in the case of the westmost German Alps population of STO (BAC, KAM, GRA and SIG: *I* = 0.347 ± 0.347; STO: *I* = 0.540 ± 0.151). The genetic differentiation measured by the pairwise *F_ST_* (see Figure 4B) revealed that the most similar population to the Cantabrian Mountains *N. pumila* population was the Massif Central population (PopAs-PopFr2: *F_ST_* = 0365), with the former being more differentiated from the Central European populations, obtaining values ranging from 0.597 in the case of the Jura population (PopAS-ABB *F_ST_* = 0.587) to 0.668 in the case of the eastmost Swiss Plateau population of GRA (PopAs-GRA *F_ST_* = 0.668). The results also revealed that the south-west isolated French population from the Massif Central was more differentiated from the Iberian isolated population from the Cantabrian Mountains than from the Central European populations, as the *F_ST_* values obtained ranged from 0.429 when compared to the Jura population (PopFr2-ABB *F_ST_* = 0.429) to 0.355 when compared to the eastmost Swiss Plateau population of GRA (PopFr2-GRA *F_ST_* = 0.547). These results indicate that the Massif Central population is distant to both the Iberian and the Central European populations. Regarding the Central European populations, the German Alps populations (STO and SIG) and the eastmost Swiss Plateau population (GRA) had little to no differentiation among each other (STO-GRA: *F_ST_* = 0.010; SIG-GRA: *F_ST_* = 0.000; and SIIG-STO: F_ST_ = 0.008), with the eastmost Swiss Plateau population (KAM) being the least differentiated population of these three populations (KAM-GRA: *F_ST_* = 0.333; KAM-STO: *F_ST_* = 0.287; and KAM-SIG: *F_ST_* = 0.333). All the German Alps and east Swiss Plateau populations were most differentiated from the Vosges population (BAC) (BAC-GRA: *F_ST_* = 0.750; BAC-STO: *F_ST_* = 0.692; BAC-SIG: *F_ST_* = 0.750; and BAC-KAM: *F_ST_* = 0.714). On the other hand, the Vosges population was less differentiated from the Austrian Alps and the Massif Central populations (HAL and PopFr2), which were less differentiated from each other (BAC-HAL: *F_ST_* = 0.456 and BAC-PopFR2: *F_ST_* = 0.467).

The PCoA (see Figure 4C), which axes explain 43.23% and 24.03% of the genetic variance, indicates the existence of three main clusters of individuals: the south-west cluster formed by individuals of the Massif Central and the Cantabrian Mountains populations (PopAs and PopFr2); the Jura–East cluster formed by individuals of the Jura, the east Swiss Plateau and the German Alps populations (ABB, KAM, GRA, SIG and STO); and the Vosges–Austrian Alps cluster formed by individuals belonging to BAC and HAL. The α-score of the DAPC, obtained after conducting a preliminary analysis in which all the possible PCs were retained, indicated that the most adequate number of PCs to be retained was six. The results of the DAPC retaining six PCs (see Figure 4D) retrieved similar groups to those of the PCoA as the south-west cluster was present, although the rest of the individuals of the central European populations presented different distributions. The Jura population (ABB) did not form part of the Jura–East cluster; instead, it formed a cluster with the Austrian Alps population (HAL). The latter was also separated from the Vosges population (BAC), which formed its own cluster. On the other hand, the east Swiss Plateau and the German Alps populations (ABB, KAM, GRA, SIG and STO) formed more or less compact cluster in which many individuals from these populations overlapped. All these results were also supported by the membership probabilities of the individuals, which revealed that the individuals of the east Swiss Plateau and the German Alps populations had similar probabilities of belonging to any of the populations forming that cluster.

**Figure 5:**
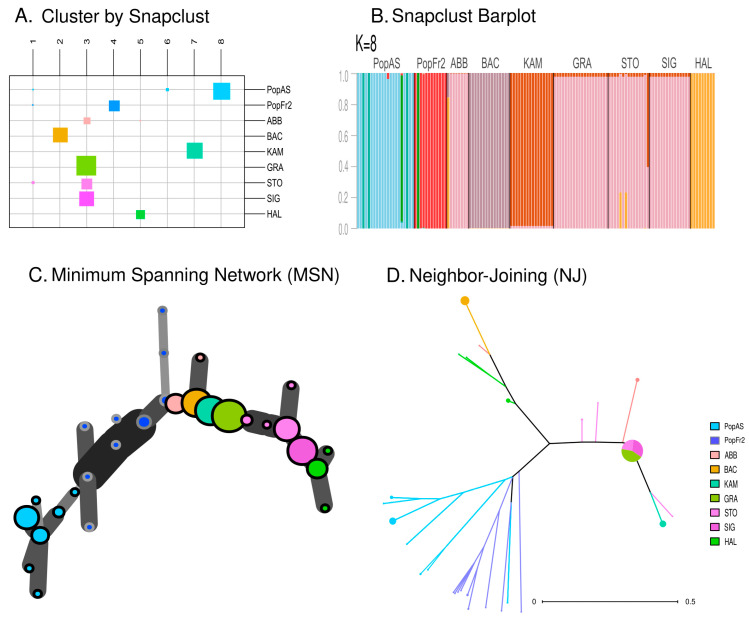
(**A**) Assignment of the individuals of each population to each of the clusters inferred using the snapclust analysis (K = 8). The size of the squares corresponds to the number of individuals attributed to each inferred cluster. (**B**) Barplot representing the membership probability of each individual belonging to each cluster in the snapclust analysis (K = 8). (**C**) Minimum Spanning Network (MSN) dendrogram based on Bruvo distance. (**D**) Neighbor-Joining network based on a dissimilarity matrix.

The ML-based clustering method using snapclust indicated that the most adequate number of clusters was around K = 8 (see Figure 5A,B). However, two of the clusters inferred using the method were formed by a few individuals of the Cantabrian Mountains population, the Massif Central population and the German Alps population of STO (see Figure 5A). This clustering method generated two specific clusters for the Cantabrian Mountains (PopAs) and the Massif Central populations (PopFr2), while grouping together the Jura population (ABB), the east Swiss Plateau population of GRA and the German Alps populations (SIG and STO) in the same cluster. On the other hand, the Austrian Alps population (HAL), the Vosges population (BAC) and the westmost KAM population of east Swiss Plateau formed their own clusters, thus showing a similar clustering for these populations to that obtained using the STRUCTURE analysis when K = 7 (Figure 3C). Regarding the networks, the MSN network (see Figure 5C) retrieved a topology that resembled the geographical distribution of the sampled populations: the different individuals of the populations were closely related to the individuals of the nearest population. On the other hand, the NJ network based on the dissimilarity matrix (Figure 5D) retrieved a topology in which three main clusters could be observed: the south-west cluster formed by the Cantabrian Mountains and the Massif Central populations (PopAs and PopFr2); the Jura–East cluster formed by the Jura, the east Swiss Plateau and the German Alps populations (ABB, KAM, GRA, SIG and STO); and the Vosges–Austrian Alps cluster formed by individuals of the HAL and BAC. This NJ clustering was similar to that obtained using the PCoA analysis, especially regarding the close relation between the Jura population (ABB) and the east Swiss Plateau and German Alps populations and the close relation between the Vosges and the Austrian Alps cluster populations (BAC and HAL).

#### 3.2.2. Spatial Analyses and Boundary Detection 

**Figure 6:**
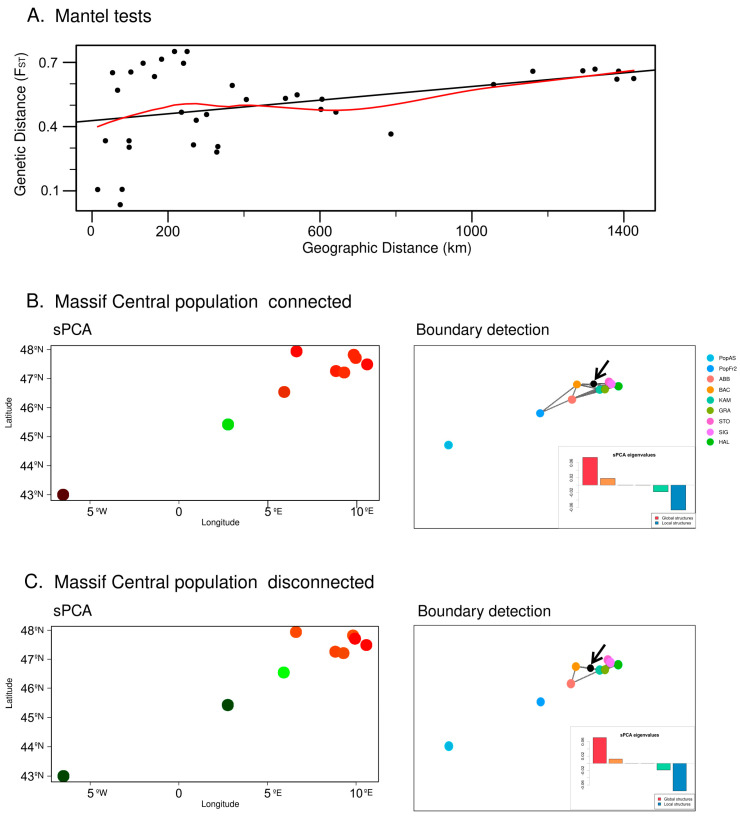
(**A**) Mantel test based on the geographical distance and the genetic distance estimated by the F_ST_. (**B**) Results of the spatial PCA (sPCA) and the boundary detection using the Monmonier algorithm, based on a connection network in which the Massif Central *N. pumila* population (PopFr2) is connected to the Alps populations. The detected boundary is represented by a black dot and is further indicated by a black arrow; on the other hand, the colors of the dots represent genetic similarity (more similar colors represent higher genetic similarity). (**C**) Results of the spatial PCA (sPCA) and boundary detection using the Monmonier algorithm, based on a connection network in which both the Massif Central *N. pumila* population (PopFr2) and the Iberian *N. pumila* population are disconnected from the Alps populations. The detected boundary is represented by a black dot and is further indicated by a black arrow; the colors of the dots represent genetic similarity (more similar colors represent higher genetic similarity).

The Mantel test (see Figure 6A) detected a slight lineal correlation between the geographical distance and the genetic distance of the populations as measured using the F_ST_ (R2 = 0.12; *p*-value = 0.02188). The two different networks used as based on the sPCA analyses, with one assuming only the disconnection of the Cantabrian Mountains population and the other assuming the disconnection of both the Cantabrian Mountains and the Massif Central populations, also suggested the existence of local and global structures (Cantabrian Mountains disconnected and Massif Central connected: Global test *p*-value = 1 × 10^−4^, r = 0.126569; Local test *p*-value = 2 × 10^−4^, r = 0.03504604. Both Cantabrian Mountains and Massif Central disconnected: Global *p*-value = 1 × 10^−4^, r = 0.1039409; Local test *p*-value = 1 × 10^−4^, r = 0.04463672). Hence, both positive and negative eigenvalues were used to estimate the sPCAs. The sPCA that assumed only the disconnection of the Cantabrian Mountains population indicated that the Cantabrian Mountains population belonged to a group separated from that of the Massif Central population, which belonged to a different group from that of the Central European populations (see Figure 6B). These results suggest the existence of a strong west–east spatial genetic structure within the studied populations. This analysis also suggests the existence of a mild spatial structure within the Central European populations. Under this scenario, the Monmonier analysis detected a boundary between the Central European populations: the discontinuity was found between the Vosges population (BAC) and the east Swiss Plateau population and between the German and the Austrian Alps populations (KAM, GRA, STO, SIG and HAL) (see Figure 6B). The sPCA based on the second scenario, which assumed the disconnection of both the Cantabrian Mountains and the Massif Central populations from the Central European populations, also revealed the existence of a strong west–east spatial genetic structure, although, in this case, the Cantabrian Mountains and the Massif Central populations belonged to the same group, with the Jura population (ABB) being in an intermediate position between these south-west groups and the rest of the Central European populations (see Figure 6C). On the other hand, under this scenario, the Monmonier analysis detected again the same boundary between the Vosges population and the east Swiss Plateau population and between the German and the Austrian Alps populations.

## 4. Discussion

The microsatellite analyses based on two markers did not clearly suggest an ongoing hybridization process between *N. pumila* and *N. lutea* in the Cantabrian Mountains, as this population exhibited a high genetic differentiation from the Massif Central *N. lutea* population. In this sense, the PCoA analysis suggested that there is an important overlap between the *N. pumila* and the *N. lutea* populations from the Massif Central—probably highly influenced by the fixation of the allele 140 at the locus NLTG/GA1 in the *N. lutea* population and the presence of the allele 133 at the locus NLGA7 in both populations— and a more restricted overlap between the two *N. pumila* populations—in this case, probably heavily influenced by the genetic composition of both populations at locus NLTG/GA1, with the allele 140 being absent in the Cantabrian Mountains population. This overlap of the Massif Central *N. pumila* population with the other two populations explains the fact that the third cluster in the less conservative STRUCTURE analysis is shared by the three populations. Nevertheless, the credibility of this third cluster is low given the tendency of the Evanno and the Puechmaille methods to overestimate the most adequate K value when there is uneven sampling [59,60], as well as the allelic composition of the Cantabrian Mountains *N. pumila* population and the Massif Central *N. lutea* population and the distance separating these two populations. This idea is further supported, on the one hand, by the obtained genetic distance, which is high between the Cantabrian Mountains *N. pumila* and the Massif Central *N. lutea* populations, and, on the other hand, by the low genetic flow between the populations as revealed by the results of the AMOVA analysis, which indicated that the three populations do not form one single group. In this sense, the hypothesis of an absence of hybridization in the isolated north Iberian population is further supported by a previous study [4], which focused on the nuclear and plastid markers of these populations and did not find traces of an ongoing hybridization process with *N. lutea*. Thus, the current results suggest that hybridization with *N. lutea* is not a conservational issue for the *N. pumila* population of the Cantabrian Mountains, unlike the potential consequence of ongoing climate change on its habitat, which has been regarded as one of the main threats to the long-term survival of this isolated population [4,38]. Additionally, these findings give further support to the observations of [5] regarding the absence of hybridization when populations are located at an altitude over 1.000 m (as is our case, since Laguna de Reconcos is over 1.600 m of altitude) and the value of these populations for the conservational effort of alpine populations.

In a wider context, the *N. pumila* population of the Laguna de Reconcos in the Cantabrian Mountains exhibits the lowest observed heterozygosity of all the studied *N. pumila* relict populations, which is in accordance with the expected features of rear-edge populations [81] and has already been observed in other relict populations in the Cantabrian Mountains, such as *Salix hastata* L. (1753) and *Juncus balticus* Willd. (1809) [82]. Interestingly, the comparison of this population with the Massif Central and Central European relict populations revealed that this north Iberian population presents several private and unique genotypes, which would give more conservational interest to this population due to its genetic uniqueness. This uniqueness is further supported by the cluster and spatial analyses, which reveal the existence of a strong spatial component in the genetic diversity of the *N. pumila* populations of the relict areas of Western and Central Europe. This has implications for the conservation of these populations at the European level since there seems to be between five and seven conservational units, with the Iberian and the Massif Central populations having two of these conservational units no matter how conservative the estimation is. Moreover, the detected signals of isolation based on genetic distance and the low gene flow detected between the Iberian and the other *N. pumila* populations suggest that the Cantabrian Mountains population could potentially be subjected to stochastic processes, which could lead to genetic decline and the extinction of this genetically unique population [81]; these findings justify its recent reassessment as being endangered [31]. Nevertheless, it should be noted that, in the Iberian context, the taxon *N. pumila* has been considered as the subspecies *Nuphar luteum* subsp. *pumilum* (Timm) Bonnier & Layens (1894) (=*Nuphar lutea* subsp. *pumila* (Timm) E.O.Beal, J. Elisha Mitchell (1956)) in recent years [1], which means that some of the Iberian *Nuphar* populations that have been registered as *N. lutea* could in fact be *N. pumila*. Hence, a morphological revision of the reported *Nuphar* populations should be considered, especially in lakes and ponds of the Pyrenees and north Iberian mountainous systems, to match the ecological references of *N. pumila* relict populations [3,5,7,8,9,10] in order to determine if the Laguna de Reconcos population is the only west relict population.

The case of the Massif Central *N. pumila* population is more complicated since the observed genetic distance, the overlap PCoA and the STRUCTURE analyses, along with the unexpectedly high heterozygosity, when compared to the Central European population suggest the possibility of an ongoing hybridization process with its neighboring N. lutea population. However, this is at odds with the results of Cires et al. [4], who found no traces of hybridization based on a ribotype and haplotype analysis. In this sense, it is difficult to discern whether this conflict is due to a limited power of discrimination of the loci proposed by Ouborg et al. [36] when it comes to these closely related species (Padgett et al., 1999), to an ongoing hybridization process as reported in the Central European relict populations by Arrigo et al. [5] and Bétrisey et al. [9], or to an ancient gene flow in the area that results in introgression as proposed by Volkova et al. [6]. To elucidate this matter, more information is needed regarding the genetic diversity and allelic composition of N. lutea and *N. pumila* populations in areas where *N. pumila* is considered relict, as well as in other areas of distribution of both species, as highlighted in [4,6]. Furthermore, the hypothesis of hybridization at altitudes over 1.000 m [5] is supported by the Cantabrian Mountains population, but not clearly by the Massif Central population (as the Lac de la Landie is at 1.039 m of altitude). In any case, the *N. pumila* relict population meta-analysis reveals that the allelic composition of the Massif Central population is different from those of the Central European populations, representing a different conservational unit.

In this sense, the *N. pumila* meta-analysis revealed that the populations of the mountainous systems of the Cantabrian Mountains, the population of the Massif Central, and the group formed by the Jura, the Swiss Plateau and the German Alps populations represent three different genetic groups. We favor the hypothesis that the Vosges and the Austrian Alps populations belong to two different genetic groups, as the various clustering methods employed in this study retrieved results—which could be greatly influenced by the fixation of the same allele at the locus NLTG/GA1—that support both hypotheses, while the boundary detection analysis suggested the existence of a discontinuity between the Vosges population and the populations at the intermediate geographical position with the Austrian Alps population. Nonetheless, more information is needed in order to determine whether there is genetic continuity joining the west Swiss Plateau with the group formed by the Jura, the Swiss Plateau and the German Alps populations or not. The Monmonier algorithm detected a boundary between the Vosges population and the group formed by the Jura and the Swiss Plateau populations and between the German Alps and the Vosges populations based on our hypothesis (the disconnection of the Iberian population from the rest of the *N. pumila* populations and disconnection of both the Iberian and the Massif Central populations). These results, together with the sPCA and the MSN results, raise new questions regarding the connection between the Western and Central Europe mountainous systems as this boundary could be caused by (1) the existence of at least two different converging colonization routes in Central Europe, with one coming from the north connecting the Vosges, the Jura and the Massif Central and one coming from the east connecting the Austrian and German Alps and the Swiss Plateau, or (2) the existence of an ancient or a recent abrupt disconnection between the Austrian and German Alps and between the Swiss Plateau and the Vosges. Thus, our understanding of the history of the Central European population would benefit from studies focusing on the genetic diversity of the north and east Europe populations.

Furthermore, studies on the long-distance dispersal mechanism of *N. pumila* would also shed light on the connectivity among the relict populations. In this context, bird endozoochory is regarded as being far less relevant than epizoochory and hydrochory for *N. lutea* long-distance dispersal [83,84]. The stepping-stone model has been suggested as a plausible long-distance dispersal model for both *N. lutea* and *N. pumila* [6,85]. However, our PCoA and DAPC analyses did not clearly support neither the stepping-stone model nor the island model as depicted by Jombart et al. [67]. This could be due to the number of microsatellites and the geographical distance between the studied populations, which means that the inclusion of more *N. pumila* populations and the use of more loci would clarify these results.

In a global context, *N. pumila* is listed in the IUCN Red List of Threatened Species [27] as Least Concern (LC). Nevertheless, although the assessment recognized the decline of *N. pumila* populations in certain parts of its range, its categorization as LC is based on it being “wide-spread” in the United States and Canada, although neither the Flora of North America nor the Database of Vascular Plants of Canada (VASCAN) report the presence of this species [86,87]. This assessment should also take into account the results of previous studies, e.g., [5,6,9,19], as well as observations regarding the relevance of wetlands and the long-time involvement of an epizoochoric bird-mediated, stepping-stone long-dispersal model [85] in the generalist *N. lutea*.

## Figures and Tables

**Table 1:** Genetic diversity parameters of the Iberian and French isolated populations. *N_A_* corresponds to the number of observed alleles per locus; *N_E_* corresponds to the effective number of alleles per locus; *H_E_* corresponds to the expected heterozygosity; *H_O_* corresponds to the observed heterozygosity; *%P* corresponds to the percentage of polymorphic loci; *I* corresponds to the Shannon Information Index; *G″_ST_* corresponds to the standardized coefficient of genetic differentiation; and *Nm* corresponds to the number of effective migrants per generation. The numerical values are accompanied by the Standard Errors.

Population	*N_A_*	*N_E_*	*H_E_*	*H_O_*	*%P*	*I*	*G″_ST_*	*Nm*
PopAS	4.500 ± 1.500	1.722 ± 0.301	0.401 ± 0.105	0.287 ± 0.237	100	0.774 ± 0.238		
PopFr1	4.000 ± 3.000	1.624 ± 0.624	0.278 ± 0.278	0.250 ± 0.250	50	0.630 ± 0.630		
PopFr2	7.000 ± 1.000	3.440 ± 0.560	0.701 ± 0.049	0.792 ± 0.042	100	1.480 ± 0.169		
Mean	5.167 ± 1.078	2.262 ± 0.438	0.460 ± 0.111	0.443 ± 0.142	83.33 ± 16.67	0.961 ± 0.244	0.808	0.515 ± 0.272

**Table 2:** Results of the AMOVA analysis of the 3 isolated populations of the Iberian Peninsula and southern France. d.f. corresponds to the degree of freedom of each analysis; SS indicates the sum of squares; MS indicates the summary of matches; Est. Var. corresponds to the estimated variation among or within populations; CV corresponds to the percentage of molecular variation explained by each variation source; *F_ST_* corresponds to the Fixation Index estimated by 9999 permutations; *F_IS_* corresponds to the Inbreeding Coefficient (within individuals) estimated by 9999 permutations; and *F_IT_* corresponds to the Total Inbreeding Coefficient estimated by 9999 permutations.

AMOVA							
Variation Source	d.f.	SS	MS	Est. Var.	CV (%)	F-Statistic	*p*-Value
Among populations	2	20.927	10.463	0.366	43%	FST = 0.427	<0.001
Among individuals	40	22.550	0.564	0.073	8%	FIS = −0.148	0.018
Within individuals	43	18.000	0.419	0.419	49%	FIT = −0.511	<0.001
Total	85	61.447		0.857	100%		

**Table 3:** Main features of the populations studied using genetic diversity meta-analysis. n corresponds to the population size; *N_A_* corresponds to the number of observed alleles per locus; *N_E_* corresponds to the effective number of alleles per locus; MLG corresponds to the number of different genotypes found in a population; MLGE corresponds to the expected MLG at the lowest common population size; *H_E_* corresponds to the expected heterozygosity; *H_O_* corresponds to the observed heterozygosity; %*P* corresponds to the percentage of polymorphic loci; and *I* corresponds to the Shannon Information Index. The numerical values of estimations are accompanied by the Standard Errors.

Population	N	*N_A_*	*N_E_*	MLG	MLGE	*H_E_*	*H_O_*	*%P*	*I*
PopAS	21	4.500 ± 1.500	1.722 ± 0.301	7	4.62 ± 0.962	0.401 ± 0.105	0.287 ± 0.237	100	0.774 ± 0.238
PopFr2	12	7.000 ± 1.000	3.440 ± 0.560	9	7.82 ± 0.649	0.701 ± 0.049	0.792 ± 0.042	100	1.480 ± 0.169
ABB	8	2.000 ± 0.000	1.640 ± 0.360	2	2.00 ± 0.377	0.359 ± 0.141	0.500 ± 0.500	100	0.535 ± 0.158
BAC	15	1.500 ± 0.500	1.500 ± 0.500	1	1.00 ± 0.000	0.250 ± 0.250	0.500 ± 0.500	50	0.347 ± 0.347
KAM	16	1.500 ± 0.500	1.500 ± 0.500	1	1.00 ± 0.000	0.250 ± 0.250	0.500 ± 0.500	50	0.347 ± 0.347
GRA	20	1.500 ± 0.500	1.500 ± 0.500	1	1.00 ± 0.000	0.250 ± 0.250	0.500 ± 0.500	50	0.347 ± 0.347
STO	15	2.500 ± 0.500	1.609 ± 0.382	4	3.24 ± 0.678	0.341 ± 0.157	0.567 ± 0.367	100	0.540 ± 0.151
SIG	15	1.500 ± 0.500	1.500 ± 0.500	1	1.00 ± 0.000	0.259 ± 0.259	0.500 ± 0.500	50	0.347 ± 0.347
HAL	9	2.000 ± 1.000	1.727 ± 0.727	3	3.00 ± 0.000	0.314 ± 0.314	0.500 ± 0.500	50	0.482 ± 0.482
Total	131	2.667 ± 0.471	1.793 ± 0.187	27	6.09 ± 1.272	0.334 ± 0.061	0.516 ± 0.109	72.22 ± 8.78	0.577 ± 0.112

## Data Availability

All data generated or analyzed during this study are included in this published article (and its supplementary information files).

## Supplementary Materials

The following supporting information can be downloaded at https://www.mdpi.com/article/10.3390/plants12091771/s1. Table S1. Main features of the loci and primers used in this study to amplify microsatellites in *N. pumila*. Table S2. Main features of the studied populations of *N. pumila*, *N. lutea* and *N. × spenneriana* from this work and previous studies [9]. Table S3. Main parameters of allelic frequencies of the Iberian and French isolated populations.
