# Supplementary material for: Genetic Diversity and Population Structure Assessed Using Microsatellite (SSR) Markers from Relict Populations of Nuphar pumila (Nymphaeaceae)
Source: Plants (Basel). 2023 Apr 26;12(9):1771. doi: 10.3390/plants12091771 (PMC10181053; doi:10.3390/plants12091771)
Supplement: Supplementary file 1 [file plants-12-01771-s001.zip › plants-2324954-SI.pdf]

# Genetic Diversity and Population Structure Assessed Using Microsatellite (SSR) Markers from Relict Populations of *Nuphar pumila* (Nymphaeaceae)

**Claudia González-Toral [1], Candela Cuesta [1,2] and Eduardo Cires [1,3,*]**

[1] Department of Organisms and Systems Biology, University of Oviedo, C/Catedrático Rodrigo Uría s/n, 33071 Oviedo, Spain
[2] Polytechnic School of Mieres (PSM), 3ª Planta Ala Sur. C/Gonzalo Gutiérrez Quirós s/n, 33600 Mieres, Spain
[3] Institute of Natural Resources and Territorial Planning (INDUROT), Campus de Mieres, C/Gonzalo, Gutiérrez Quirós s/n, 33600 Mieres, Spain
* Correspondence: cireseduardo@uniovi.es; Tel.: +34-104780

**Table S1**. Main features of the loci and primers used in this study to amplify microsatellites in *N. pumila.*

| Locus | Repetitive motive | Range (Pb) | Primers sequence ( 5'-3') | Reference |
|---|---|---|---|---|
| NLTG/GA1 | (GT)8 (TG)14 (GA)9 | 118–140 | F: AAGCAGCAGCAAAATTTGTA<br>R: TGTGCAAGTTACCTGTTTCC | [36] |
| NLGA7 | (GA)24 | 80–108 | F: ATTTATTCCCAGCACTTTGG<br>R: CTTGACATGATTTCTCTGAACC | [36] |

**Table S2**. Main features of the studied populations of *N. pumila*, *N. lutea* and *N. × spenneriana* from this work and previous studies [9]. The table specifies the code name of the population (Pop Code), the locality (including water source, whenever possible to give a specific name, locality and country), the number of *Nuphar* taxa present (+), the total number of individual (Tot. Ind.) and the range of alleles found for each marker (NLGA7, NLTG/GA1) as number of repetitions. *= fix alleles.

| Pop Code | Locality | *Nuphar lutea* | *Nuphar pumila* | *Nuphar × spenneriana* | Tot. Ind. | NLGA7 range | NLTG/GA1 range |
|---|---|---|---|---|---|---|---|
| FIL | Filzweiher, Tutzing (Germany) | | | + | 15 | 58-104 | 117-135 |
| HAL | Haldensee, Haller (Austria) | | + | | 14 | 64-81 | 131* |
| KES | Kesselsee, Bad Endorf (Germany) | + | | | 15 | 58-104 | 119-123 |
| ROH | Görisried (Germany) | | + | + | 16 | 64-94 | 125-133 |
| STI | Weiher Oy-Mittelberg (Germany) | + | + | | 7 | 66-83 | 117 and 133 |
| SIG | Schlossweiher, Argenbühl (Germany) | | + | | 15 | 64 and 81 | 133* |
| STO | Stockweiher, Wolfegg (Germany) | | + | | 15 | 64 and 81 | 131-135 |
| SCH | Schlüchtsee, GrafenGausen (Germany) | | | + | 13 | 64-104 | 121 and 133 |
| BGF | Fribourg (Switzerland) | | + | | 9 | 62 and 81 | 133* |
| LAU | Lausanne (Switzerland) | + | | | 3 | 90** | 121** |

| Pop Code | Locality | *Nuphar lutea* | *Nuphar pumila* | *Nuphar* × *spenneriana* | Tot. Ind. | NLGA7 range | NLTG/GA1 range |
|---|---|---|---|---|---|---|---|
| JON | Lac des Joncs, Châtel-Saint-Denis (Switzerland) | | + | | 4 | 62 and 81** | 133** |
| LUS | Lac de Lussy, Châtel-Saint-Denis (Switzerland) | | | + | 28 | 62-90 | 121 ad 133 |
| ABB | Lac de l'Abbaye, Grande-Rivière (France) | | + | + | 9 | 62-81 | 131 and 133 |
| BSG | St. Gall (Switzerland) | | | + | 11 | 62 and 85 | 66 and 121 |
| ZUA | Weinegg, Zurich (Switzerland) | | + | | 6 | 64 and 81** | 133* |
| GRA | Gräppelensee, Alt Sankt Johann (Switzerland) | | + | | 20 | 64 and 81 | 133* |
| BAC | Étang Bachetey, La Rosière (France) | | + | | 15 | 62 and 79 | 131* |
| ZUB | Weinegg, Zurich (Switzerland) | | + | | 13 | 62 and 79 | 133* |
| KAM | Bubikon (Switzerland) | | + | + | 25 | 64-90 | 123-135 |
| UST | Zurich (Switzerland) | | + | | 3 | 64 and 79** | 133** |
| WAN | Zurich (Switzerland) | | + | | 3 | 64 and 79** | 133** |
| PopAS | Laguna de Reconcos, Riomolín (Spain) | | + | | 21 | 115-134 | 115-146 |
| PopFr1 | Lac de Laspiadales, Saint-Genès-Champespe (France) | + | | | 10 | 101-134 | 140* |
| PopFr2 | Lac de La Landie, Saint-Genès-Champespe (France) | | + | | 12 | 100-152 | 118-172 |

**Table S3**. Main parameters of allelic frequencies of the Iberian and French isolated populations.

| Population | $N_A$ | $N_A$ (≥5%) | $N_E$ | No. Private alleles |
|---|---|---|---|---|
| PopAS | 4.500 ± 1.500 | 2.000 ± 0.000 | 1.722 ± 0.301 | 2.000 ± 0.000 |
| PopFr1 | 4.000 ± 3.000 | 4.000 ± 3.000 | 1.624 ± 0.624 | 0.500 ± 0.500 |
| PopFr2 | 7.000 ± 1.000 | 3.000 ± 0.000 | 3.440 ± 0.560 | 4.000 ± 0.00 |
